# Development of a theory- and evidence-based intervention to enhance implementation of physical therapy guidelines for the management of low back pain

**DOI:** 10.1186/2049-3258-72-1

**Published:** 2014-01-15

**Authors:** Geert M Rutten, Janneke Harting, Leona K Bartholomew, Jozé C Braspenning, Rob van Dolder, Marcel FGJ Heijmans, Erik JM Hendriks, Stef PJ Kremers, Roland PS van Peppen, Steven TJ Rutten, Angelique Schlief, Nanne K de Vries, Rob AB Oostendorp

**Affiliations:** 1 NUTRIM, Department of Health Promotion, Maastricht University; Faculty of Health, Medicine and Life Sciences, P.O. Box 616, Maastricht 6200, MD, The Netherlands; 2 Department of Public Health, Academic Medical Centre University of Amsterdam, PO BOX 22660, Amsterdam 1100, DD, The Netherlands; 3 Division of Health Promotion and Behavioral Sciences, School of Public Health, University of Texas Health Science Center at Houston, 1200 Herman Pressler Blvd., Houston 77030, TX, USA; 4 Radboud University Nijmegen Medical Centre, Scientific Institute for Quality in Healthcare (IQ healthcare), Nijmegen 6500, HB, The Netherlands; 5 Department of Physiotherapy and Research Centre for Innovation in Health Care, Institute for Human Movement Studies, University of Applied Sciences, PO Box 85182, 3508 AD, Utrecht, The Netherlands; 6 Fysiomaatwerk Heeswijk Dinther, Physical Therapy and Manipulative Physical Therapy, Heilarensestraat 64a, Heeswijk Dinther 5473, RB, The Netherlands; 7 Department of Epidemiology, Maastricht University; Faculty of Health, Medicine and Life Sciences, PO Box 616, Maastricht 6200, MD, The Netherlands; 8 Therapeuticum Aachen Laurensberg, Physical Therapy and Manipulative Physical Therapy, Schurzelter Str. 25, Aachen 52074, Germany; 9 FH Aachen University of Applied Sciences, Heinrich-Mußmann-Str. 1, Jülich 52428, Germany

**Keywords:** Guideline implementation, Low back pain, Quality improvement, Multilevel program, Individual professional, Practice management, Physical therapy

## Abstract

**Background:**

Systematic planning could improve the generally moderate effectiveness of interventions to enhance adherence to clinical practice guidelines. The aim of our study was to demonstrate how the process of Intervention Mapping was used to develop an intervention to address the lack of adherence to the national CPG for low back pain by Dutch physical therapists.

**Methods:**

We systematically developed a program to improve adherence to the Dutch physical therapy guidelines for low back pain. Based on multi-method formative research, we formulated program and change objectives. Selected theory-based methods of change and practical applications were combined into an intervention program. Implementation and evaluation plans were developed.

**Results:**

Formative research revealed influential determinants for physical therapists and practice quality managers. Self-regulation was appropriate because both the physical therapists and the practice managers needed to monitor current practice and make and implement plans for change. The program stimulated interaction between practice levels by emphasizing collective goal setting. It combined practical applications, such as knowledge transfer and discussion-and-feedback, based on theory-based methods, such as consciousness raising and active learning. The implementation plan incorporated the wider environment. The evaluation plan included an effect and process evaluation.

**Conclusions:**

Intervention Mapping is a useful framework for formative data in program planning in the field of clinical guideline implementation. However, a decision aid to select determinants of guideline adherence identified in the formative research to analyse the problem may increase the efficiency of the application of the Intervention Mapping process.

## Background

Adherence to clinical practice guidelines (CPGs) is limited, and interventions to enhance uptake have been only moderately effective [1,2]. This could be because, despite the increased attention to behavioural and organizational theory application in implementation science, few interventions to improve adherence are based on a coherent theoretical framework and formative research [3,4]. Intervention development should include formative research to provide an analysis of influential barriers to and facilitators of CPG implementation and a deliberate matching of theoretical behavioural and environmental change methods to these factors [5,6].

Non-specific low back pain constitutes a serious public health problem associated with significant socioeconomic burden, and physical therapy is expected to contribute to the reduction or elimination of this burden [7]. Although only 2-7% of the patients with acute low back pain develop chronic low back pain, recurrent and chronic low back pain account for 75-85% of total worker’s absenteeism. To support physical therapists as they manage patients with low back pain, the Royal Dutch Association for Physical Therapy developed a national physical therapy [8] and a separate manual therapy CPG [9]. The guidelines urge clinical reasoning, assessment and management of psychosocial factors, and documentation including outcome measurement. Their four main features are: applying the International Classification of Functioning, Disability and Health (ICF); identifying and applying patient profiles with duration, course, and psychosocial factors influencing recovery; restricting the application of manipulative physical therapy and limiting the number of treatment sessions; and focusing on patient behaviour to restore physical activity and social participation. Previous studies support the assumption that greater adherence to CPGs for low back pain provide a cost advantage [10,11], and a recent study related guideline adherence to improved physical functioning [12].

The aim of our study was to demonstrate how the process of Intervention Mapping [13]can be used to develop an intervention to address the lack of adherence to the national CPG for low back pain by Dutch physical therapists [14,15]. After its development, the intervention was evaluated on its feasibility and potential effectiveness in a pilot test of which the results are reported elsewhere [16].

### Formative research and program development

#### Program planning team and procedures

The three core project team members, a doctoral candidate, faculty member and project coordinator, performed the formative research and initial program development. Advisory group members, including the leader of the Dutch physical therapy guidelines program, a member from the Royal Dutch Association for Physical Therapy responsible for quality policy, practicing physical therapists, and a representative of an interest group on patient and healthcare provider communication, began working on the project early in the intervention development phase.

### Formative research

#### Formative research methods

Based on the specific recommendations in the CPGs for low back pain, we first developed a set of indicators to operationalize guideline adherence [12]. Then, we focused on the limited adherence of Dutch physical therapists [14,15]. We used a multimethod approach to understanding the behavioural and environmental factors that influence guideline adherence [17], consisting of two literature reviews and a series of theory-based qualitative [18] and quantitative studies [15] (for detailed information see Additional file 1). In the first literature review we made an inventory of individual health care providers’ cognitive factors related to guideline adherence. Three focus group interviews (n = 30) were held to make these factors specific for physical therapy. The subsequent cross sectional survey (n = 472), resulted in quantitative data, which allowed us to assess the strength of the relation between these cognitive factors and guideline adherence. In the second literature study we included affective and organizational factors related to guideline adherence. Four additional focus group interviews (n = 29) were held to assess the relevance of these factors to physical therapy. Finally, we conducted a longitudinal survey (n = 394) to determine which cognitive, affective and organizational factors explained and predicted guideline adherence.

### Formative findings

**Figure 1:**
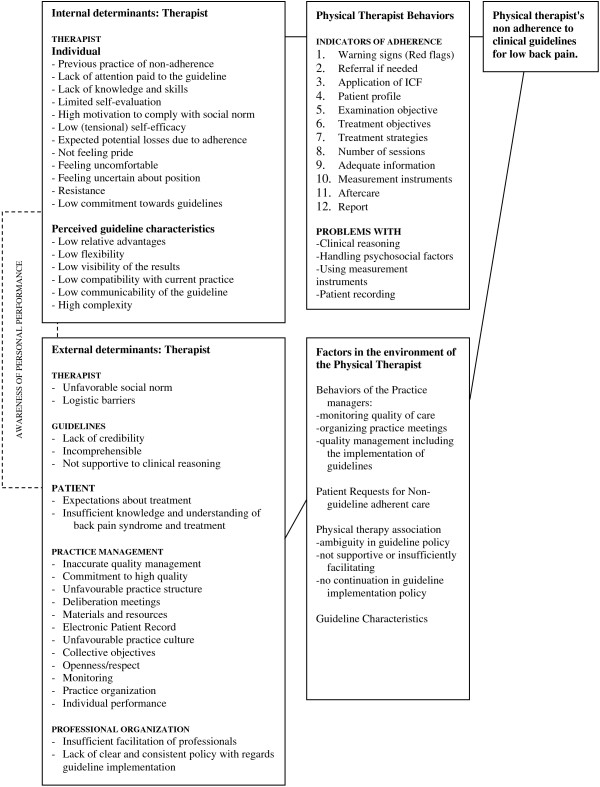
Logic model of factors associated with non-adherence to guidelines for non-specific low back pain.

We used the results of our multi method formative work to develop a synthesis of most important determinants. Subsequently, we organized our findings into a logic model of the problem of lack of guideline adherence highlighting the central roles of therapists and the practice quality managers (see Figure 1). This model was presented to and discussed with the members of the program planning team to check if the model actually covered the most important determinants.

Based on the guidelines, we described adherence with 12 individual indicators from the guidelines, they are: 1. assessing warning signs of the need for physician referral, 2. making a physician referral if needed 3. applying the ICF, 4. assessing a patient profile, 5. choosing examination objectives based on the profile, 6. creating treatment objectives based on the profile, 7. developing treatment strategies based on the profile; 8. determining maximum number of treatment sessions, 9. providing adequate patient information; 10. measuring outcomes, 11. arranging aftercare, 12. providing a written report to the referring physician [16].

We found the most important personal influences on physical therapists’ performance were paying attention to the guidelines, knowing content, feeling comfortable and certain about adherent care, perceiving guideline characteristics positively, expecting positive outcomes from adherence, having sufficient self-efficacy and skills to apply adherent care, having a positive social norm regarding adherence and experiencing little motivation to comply with patients who prefer non-adherent care. The personal determinants of the behaviour of quality managers were knowledge of quality management, commitment and a positive attitude towards high-quality care, positive social influences with respect to quality management, self-efficacy and skills with regard to management and monitoring tasks, and motivation and advocacy skills.

Environmental influences for the therapists included adverse social norm and barriers. The CPGs on low back pain were judged by some to lack credibility, to be incomprehensible, and to hamper clinical reasoning. Practice management characteristics included inaccurate quality management, unfavourable practice culture, and lack of monitoring. The professional association was seen as not providing sufficient facilitation and as lacking a clear and consistent policy with regard to guideline implementation. Patients also play a significant role in the environment of therapists’ adherence when they lobby for hands-on and extended care. These demands were related to patients’ inadequate understanding of the natural course of low back pain, inappropriate expectations of the physical therapy treatment, and insufficient information about the role of psychosocial factors.

## Methods

### Intervention mapping

Following the Intervention Mapping framework, we completed the following program development steps based on our formative findings. The project team focused the intervention on two interacting practice levels: private practice physical therapy and practice quality management. Due to issues of quality control and quality certification by health insurance companies, there is a growing tendency in Dutch physical therapy practices to make one of their colleagues responsible for quality management.

### Change objectives

In the first three months of the Intervention Mapping process, we created matrices of change objectives. Change objectives combine the expected performance of our two groups of proposed intervention participants, physical therapists and quality managers, with determinants that describe influences on performance. We based the performance objectives on the effect of the practice level (quality manager behaviour) on physical therapist adherence; the 12 quality indicators that reflect guideline adherence; formative findings regarding the importance of clinical reasoning (dealing with psychosocial factors, using outcome measurement instruments, and recording patient data) and a guiding theory of individual and organizational learning self-regulation (self-reflection, self-judgment, goal setting, planning and action) [19,20].

#### Performance objectives

Performance objectives are listed in Table 1. Examples of performance objectives for clinical reasoning were choosing the correct patient profile, administering questionnaires, and adopting a hands-off approach in the case of acute low back pain with a favourable natural course. In relation to self-regulation, the physical therapists were to regularly reflect on the content of their work, judge their actual performance and react on the basis of that assessment. For quality managers, performance objectives included initiating a quality improvement project in their practice. Planning, preparing and managing the quality improvement project should include establishing a practice structure and a practice culture that facilitate guideline adherence.

**Table 1:** Performance objectives for physical therapist and practice quality managers

**Individual physical therapist**	
Overall guideline adherence	1. See the guideline as a valuable quality tool
	2. Decide to make an effort to improve their adherence to the guidelines
	3. Keep patient records that contain sufficient information to enable reflection on the quality of their work
Self regulation and goal setting	Set goals and make plans, using self-monitoring, self-judgement, self-reaction, self-evaluation and maintenance of procedure
	4a. Improve work quality by means of self-regulation
	4b. Regularly reflect on work content (self monitoring)
	4c. Judge personal performance
	4d. React on the basis of judgement
	4e. Evaluate the effect of actions
	4f. Maintain this procedure
Clinical reasoning diagnostics	5a. Correctly and completely assess the patients’ complaints in all the subsets of the ICF
	5b. Categorize the patient correctly on the basis of episode duration, course and the presence of psychosocial variables (choose the correct patient profile)
	5c. Choose adequate examination objectives and examination strategies
Clinical reasoning questionnaires	5d. Apply questionnaires
Clinical resoning treatment plan	6a. Choose applicable treatment objectives and treatment strategies
	6b. Apply a hands off approach in the case of acute LBP with a normal course 6c. Apply a limited number (maximum 4) of treatment sessions in case of acute LBP with a normal course
	6d. Provide adequate advice to the patient
	6e. Formulate sound arguments when they diverge from the guideline recommendations
Psychosocial (ps) factors	7a. Assess psychosocial factors
	7b. Integrate factors in the treatment-plan and decide about how to deal with these factors
	7c. Address factors in the treatment of the patient with low back pain
**Practice quality manager**	
Quality management	1. Decide to start a quality improvement project
	2. Plan and make preparations for a quality improvement project
	2.1 Provide the necessary materials and means for optimal quality of care
	2.2 Involve experts, if applicable
	2.3 Develop or maintain a practice culture of openness and mutual respect
	3. Manage the quality improvement project
	3.1 Bring the available materials to the attention of colleagues
	3.2 Guard the open practice culture
	3.3 Bring the possibility of cooperation with other disciplines to the attention of colleagues
	3.4 Support colleagues in their deliberation/cooperation with other relevant disciplines
	3.4 Assure the possibility for retraining
	4. Evaluate the quality improvement project
	5. Ensure care of continuation

#### Creating change objectives

We combined performance objectives with determinants to create matrices of change objectives, the specific targets for the intervention. Excerpts of the matrices are included in Table 2, and examples of change objectives are included in this section. For physical therapists to decide to improve adherence, social norms and self-efficacy were important determinants. The intervention, therefore, would have to help therapists “Recognize that patients are not extremely negative about the hands off policy or the activating approach” and “Express confidence in applying guideline adherent care even when the patient prefers non-adherent care”. At the practice management level, change objectives were related to knowledge, self-efficacy and skills for general management, monitoring, motivation and advocacy. The quality manager intervention would therefore have to bring managers to “Name and explain the steps of a quality improvement plan”, “Express confidence in developing and preparing for a quality improvement project” and “Demonstrate skills in the ability to involve colleagues in the setting of attainable goals”.

**Table 2:** Change objectives for the individual physical therapist (PT) and practice quality manager (PQM; selection)

**Determinants performance objectives**	**Knowledge (K)**	**Affective factors (AF)**	**Attitude (ATT)**	**Social influence (social norm /social support (SN)**	**General self-efficacy/skills (SES)**	**Self-efficacy/skills monitoring (MO)**	**Motivation/advocacy Skills (MA)**
**Physical therapist**							
OVERALL ADHERENCE		AF2.1 Acknowledge that the GL can evoke feelings of pride when their actual practice meets the recommendations	ATT2.1 Confirm the benefit of the GL as a knowledge document and a frame to evaluate personal performance	SN2.1 Talk about how their colleagues and physicians think about the GL	SES2.1 Express confidence in applying guideline adherent care even when the patient prefers non adherent care		
2. Decide to make an effort to improve their adherence to the GL							
		AF2.2 Acknowledge that the GL can enhance their feelings of confidence when they communicate the treatment plan with the patient		SN2.2 Recognize that patients are not extremely negative about the hands off policy or the activating approach	SES2.2 Express how they apply GL adherent care when their colleagues do not.		
SELF REGULATION AND GOAL SETTING	K4.1 Explain the principles of self regulation with respect to the quality of their work	AF4.1 Recognize the affective reactions the GL evokes in them	ATT4.1 Express and discuss the importance they attach to the GL (quality tool; evidence based practice)	SN4.1 Acknowledge that the professional association approves of using the GL	SES4.1 Describe a plan for dealing with feelings of discomfort due to self-monitoring		
4. Set goals and make plans using self-monitoring, self-judgement, self-reaction, self-evaluation and maintenance of procedure	K4.2 Explain a strategy to thoroughly reflect on the content of their work	AF4.2 Describe their affective reaction related to attainment of higher adherence rates		SN 4.2 Describe that the use of GL is becoming the practice ( social) standard	SES4.2 Express confidence in managing feelings of discomfort		
QUESTIONNAIRES	K5d.1 Distinguish the purposes for which questionnaires can be applied	AF5d.1 Recognize why questionnaires evoke feelings of discomfort	ATT5d.1 Express the belief that questionnaires support diagnostics; prognostics; effectiveness assessment; and communication with the patient	SN5d.1 Adduce arguments for the application of questionnaires	SES5d.1 Express confidence in the application of questionnaires despite the available time		
5d. Apply questionnaires							
	K5d.2 Give their interpretation of the questionnaires in the GL		ATT5d.2 Acknowledge the benefit of questionnaires for monitoring effectiveness	SN5d.2 State that it is preferable to use questionnaires	SES5d.2 Explain how they motivate their patient to complete questionnaires		
PSYCHOSOCIAL (PS) FACTORS	K7a.1 Name the PS factors that have proven to impede recovery or play a role in transition to chronic LBP and how they do that		ATT7a.1 Acknowledge the importance of the assessment of PS factors		SES7a.2 Explain how they recognize PS factors during history taking		
7a. PTs assess psychosocial factors							
	K7a3 Describe how to effectively elicit PS factors		ATT7a.2 Recognize the important role of questionnaires in the assessment of PS factors		SES7a.4 Express their confidence in the interpretation of questionnaire outcomes		
**Practice quality manager**							
2. Plan and make preparations for a quality improvement project	K2.1 Name and explain the steps of a quality improvement plan		ATT2.1 Demonstrate conviction to bring quality improvement/GL adherence to the attention of colleagues	SN2.1 Describe optimal quality of care as the practice standard	SES2.1 Express confidence in developing and preparing for a quality improvement project	MO2.1 Express confidence in the ability to identify and use (an) opinion leader(s) in the practice (if applicable)	MA2.1 Demonstrate ability to deliberate with colleagues about the ‘desired future vision’ to motivate the change
2.1 Provide the necessary materials and means for optimal quality of care			ATT2.2 Express the importance of assessing the individual ideas about/needs for quality improvement with colleagues	SN2.2 Emphasize the importance of transparency of quality for patients and health insurance companies	SES2.3 Demonstrate the ability to decide when and how to start the quality improvement project	MO2.4 Decide about the purchase and use of an EHR to monitor the quality of care	MA2.2 Demonstrate skills in ability to involve colleagues in the setting of attainable goals
				SN2.3 Show engagement in making quality improvement a collective objective in the practice			MA2.3 Express confidence to deliberate with colleagues about the time investment
3. Manage the quality improvement project							
3.1 Bring the available materials to the attention of colleagues			ATT3.1 Show enthusiasm about the quality improvement project	SN3.1 Engage in coaching and supporting (problem analysis; counseling) colleagues in case of problems or resistance	SES3.1 Explain how he is going to manage the quality improvement project	MO3.1 Demonstrate how monitoring of the quality improvement by means of the monitoring materials (patient record audits and feedback; EHR)	

GL = Guideline; EHR = Electronic Health Record.

### Theory-informed behavior change methods and practical applications

Based on the change objectives, we used the next 3–4 months to match theory-informed intervention methods to the change objectives for therapists and managers and to formulate practical applications. An intervention method (also referred to as behavior change technique [21]) is a theoretically and empirically supported process for effecting behavior change in individuals, groups, or social structures. A practical application is the way a method is delivered to match the context of the priority population.

An overview of methods and applications with reference to the theories from which they are derived is presented in Table 3. For example, from Goal Setting theory [22], we asked physical therapists to formulate goals that were challenging, moderately complex, specific, measureable, realistic and acceptable. In order to increase the likelihood of goal attainment, in addition, participants added specific plans (implementation intentions) for carrying out their goals [23].

**Table 3:** Overview of planned methods and applications

**Theories**	**Theoretical method**	**Determinants**	**Practical applications**	**Objective**
• Active learning theory [24,25]	• Information transfer	• Knowledge	Individual PT and PQM	Individual PT and PQM
			• Brief lectures	• Get acquainted with self-regulation
				• Knowledge about the content of the guidelines and measurement instruments
				PQM
				• Get acquainted with management process
				• Get acquainted with management tools
• Elaboration likelihood	• Active information processing	• Knowledge	Individual PT and PQM	Individual PT and PQM
Model [26]	• Cooperative learning	• Attitude (guideline characteristics and affective determinants)	• Small group sessions with peers and practice	• Attitude building about guideline – what does the patient gain?
• Active learning theory [24,25]	• Environmental re- evaluation	• Social norms	• Plenary discussions	• How do colleagues think about the guideline?
• Transtheoretical model [27]	• Social influence			• Better processing of new knowledge
• Theory of planned behavior [28]	• Discussion			
• Self regulation [19,29]	• Self monitoring	• Awareness	Individual PT and PQM	Individual PT
• Transtheoretical model [27]	• Conciousness raising		• Home-work assignment	• Comparing a patient record with the recommendations in the guideline for low back pain
• Precaution adoption				PQM
Process model [30]	• Personalizing ‘risk’			• Assessment of practice organization and practice change culture
	• Organizational reflection			
• Goal setting theory [22]	• Goal setting	• Outcome	Individual PT	Individual PT and PQM
	• Participation	• Expectations	• Home work assignment	• Choosing points for improvement
	• Cooperative	• Self-efficacy	• Small group work with peers	• Formulate SMART individual and collective goals
	• learning	• Intention		PQM
	• Discussion	• Commitment	Individual PT and PQM	• Leading a meeting to set goals for improvement
			• Small group work with practice	• Setting SMART collective goals
	• Feedback	• Skills	• Plenary discussion with peer and expert feedback	
•Implementation intentions [23]	• Implementation intentions	• Outcome expectations	Individual PT	Individual PT
			• Develop a personal	• Describe the SMART goals and the strategies to achieve them
•Active learning theory [24,25]	• Planning coping responses	• Self-efficacy	• Development plan (PDP)	
		• Intention	PQM	PQM
	• Feedback	• Skills	• Develop a practice quality improvement plan (PQIP)	• Describe the SMART goals and the management steps to take to achieve them
			Individual PT and PQM	• Describe the necessary means
			• Plenary presentation with peer and expert feedback of	• Estimate the costs
			the PDP’s and the PQIP’s	• Make a risk analysis
•Active learning theory [24,25]	• Active learning	• Self-efficacy	Individual PT and PQM	Individual PT
			• Home-work assignment with expert and peer feedback	• Implement one of your goals
•Social constructivism [31,32]	• Guided practice	• Skills		PQM
				• Achieve quick wins
Self regulation [19,29]	• Evaluation	• Skills	Individual PT and PQM	Individual PT and PQM
	• Organizational diagnosis/monitor-ring		• Home-work assignment	• Evaluate if the intended change was achieved and why (not)
			• Small group work with peers	
•Goal setting theory [22]	• Action planning	• Commitment	Individual PT and PQM	Individual PT and PQM
•Implementation intentions [23]	• Participation	• Intention	• Home-work assignment	• Make a plan for continuation of the process
			• Small group work with practice	• How do colleagues deal with barriers for implementation?
			• Plenary presentation with peer and expert feedback	
•Social cognitive theory [33]	• Vicarious learning	• Self-efficacy	Individual PT and PQM	Individual PT and PQM
	• Modeling	• Skills	• Meet the expert session	• Improve self-efficacy and skills about handling psychosocial factors

PT = Physical Therapist; PQM = Practice Quality Manager; SMART = Specific, Measurable, Acceptable, Realistic, Time specific.

## Results

### Program development

#### Program components

The project team proposed a series of interactive workshops, away from the worksite, as the primary intervention component to encourage peer education and for interaction between physical therapists and quality managers. Also, by participating in the workshops, the physical therapists and quality managers would break with their daily context and routines, a disruption which was expected to help them change habitual behaviour.

During the next 4 months, the project team worked with two experienced physical therapy trainers with expertise in quality improvement projects. The team provided the trainers with program, performance, and change objectives, the theory-informed behaviour change methods and practical applications, and four case descriptions of patients with low back pain. The trainers integrated these elements into a coherent and feasible program with review from the project team.

#### Program description

The program comprised six meetings: four 3-hour sessions for physical therapists and quality managers together and two 3-hour sessions for quality managers. We expected the extensive opportunity for therapist and manager interaction to enhance the quality improvement process. During the sessions, physical therapists assessed personal adherence to the guidelines by comparing a patient record with the recommendations in the guidelines. Subsequently, they chose and considered an implementation strategy for three specific, measurable, acceptable and realistic objectives for personal improvement. Trainers challenged the therapists to implement one of their objectives and to evaluate what changed in their process of care. Finally, therapists thought about how to maintain their changes. Results of these activities were a Personal Development Plan that contained pointers for individual quality improvement, goals achieved, goals still to be achieved, intended strategies, and a sustainability plan. Additionally, the physical therapists chose three collective goals with colleagues and the quality managers from their practices.

During two sessions, trainers taught the quality managers how to use a management scan to assess issues related to improvement of five organizational domains: leadership, strategy, management of means, people management, and process management [34]. Trainers also showed the managers how to assess the organizational change culture with the Personal Change Style questionnaire [35] and how to perform a Strengths, Weaknesses, Opportunities and Threats (SWOT) analysis [36]. In addition, the quality managers selected change activities for their practices and made a risk assessment and a cost analysis for the change process. Finally, the managers developed a plan to continue their quality management. Trainers asked the quality managers to find ‘quick wins’, goals that would be attainable in a short time with relatively low effort. Using the activities in the workshops, participants developed a Practice Quality Improvement Plan (PQIP) that contained quality improvement goals, intended results, outcomes of the program’s organizational analyses, chosen strategies, requirements, possible barriers and an expense estimation.

Finally, the program made use of the most current draft revision of the Dutch physical therapy CPG on low back pain (unpublished manuscript). To explicitly support clinical reasoning, this revised guideline links recommendations to findings from evaluation steps in the process of care. The workshop also provided a patient information leaflet on guideline adherent care to support physical therapists’ as they managed patient’s treatment expectations.

### Program implementation planning

From the beginning of the planning process, we paid attention to capacity for the program’s adoption, implementation and sustainability, including its practical acceptability and feasibility throughout development. The plan components to facilitate adoption, implementation and sustainability were directed at policy of the professional association to bring a focus on quality improvement; information for the patient to discourage seeking hands-on treatment and to increase awareness of the importance of psychosocial factors in low back pain; revision of the guidelines to increase support for clinical reasoning and for dealing with psychosocial factors; regular inclusion of our program in nationwide training programmes.

### Evaluation plan

The evaluation plan concerned the pilot test of the quality improvement program. Aims of the evaluation were to assess the potential effectiveness of the program as well as to evaluate the fidelity, acceptability and feasibility of the program’s implementation in an accompanying process evaluation.

For the effect evaluation, we planned a one-group pre-test/post-test study (N = 8 practices, including 30 physical therapists 8 of whom were also the quality managers of the practices). We measured adherence to the CPGs on low back pain with clinical vignettes that addressed the previously mentioned 12 indicators reflecting the guidelines’ main recommendations. These vignettes were based on validated vignettes from a previous study, which showed to have acceptable validity (Spearman’s rs = .31) to measure PTs’ guideline adherence [37-39]. Clinical reasoning was measured by assessing the consistency of physical therapists’ choices over three separate quality indicators. Consistency in choices was operationalised as the presence of the conditional argument” (if-then connective) which is an important component of human reasoning [40] (e.g. if the therapist found psychosocial factors that influence the course of recovery, than he should integrate them into the treatment plan). We measured changes in practice quality management with observations, group interviews, and document analyses, with a focus on self-regulation, commitment to quality management, transfer of knowledge to the practice, patient recording, regular deliberation meetings, patient outcome measurement, monitoring systems, and structures for sustainability. Further details as well as the results of the evaluation study are written elsewhere [16].

The process evaluation was an observational study of fidelity, acceptability and feasibility. Concerning fidelity, evaluation addressed whether the implementation addressed the planned behaviours, methods and practical applications, performance objectives and change objectives, program components and activities previously specified. We also assessed the extent to which physical therapists and quality managers participated in activities. Regarding acceptability we asked the participants’ to evaluate the intervention and materials used. Feasibility questions addressed potential barriers, such as time and financial limitations.

## Discussion

### Summary

This study demonstrates how the framework of Intervention Mapping can be used to develop interventions that aim to improve guideline adherence based on formative research. Findings from multi-method formative research provided the foundation for a logic model of the problem of physical therapists’ low adherence to clinical guidelines for low back pain. This logic model enabled the planners to first decide “who” and “what” should change as a result of the intervention. From the formative work, we decided that the intervention should influence both therapists and managers at the practice level and that these participants should actively plan and implement practice change through a process of self-regulation with therapists monitoring and analysing personal adherence, setting goals for improvement, implementing plans and evaluating outcomes. Quality managers were taught to plan and implement change at the practice level.

Based on the findings from the formative work regarding the behaviours necessary to implement clinical guidelines and the influences on behaviour at both the therapist and practice levels, we produced matrices of change objectives, the guiding documents for program development. The change objectives pointed to the selection of theory-informed behaviour change methods and practical applications and, finally, to the creation and delivery of a coherent program.

### Lessons learned

To systematically develop a multilevel intervention using the Intervention Mapping framework presented some challenges. We were surprised at the analysis needed and the lack of clear guidance for how to sort through the formative findings to select the most important performance objectives and behaviours for both the therapist and the practice levels. The formative data, including literature review, did not (and usually does not) provide clear evidence of causation - either about what people should do to reach the behaviour change targeted by the intervention, nor why they would engage in the performance when specified. For example, from the survey we found that almost 80% of the participating physical therapists were inclined to follow the patient’s preferences. This suggested to us that motivation to comply may be a salient determinant of guideline adherence, but the relation certainly could not be depicted as causal. Nevertheless, this partial evidence is generally the state of the knowledge, in terms of developing interventions and adapting evidence-based interventions to new settings. We would argue that an imperfect systematic process of selecting and using information about behaviours and determinants is much better than simply avoiding the evidence altogether. Several selection procedures have been described by other researchers [41-43], but to our knowledge no consensus exists about a preferred procedure.

A second challenge was related to the selection of theory-based behaviour change methods and practical applications. Although many theories postulate how change in behaviour may occur, the evidence for the underlying assumptions is still under development [5,21]. A recently developed model by Michie and colleagues (2011) that links behavioural conditions (that can be seen as determinants) to intervention strategies and policy categories [44] has not yet been extensively evaluated. The Intervention Mapping developers have also provided a series of tables that make suggestions and provide evidence for change methods that are matched to specific determinants and supported by examples and evidence [13]. Nevertheless, thinking about what methods might be both feasibly delivered and powerful enough to produce change can be daunting, and at this time combines both creativity and evidence.

A third issue arised while developing the intervention program. Preferably, this program would adequately address all selected determinants for optimal change [5,21]. This goal must be carefully balanced with acceptability and feasibility of the program. This tension may result in ineffective programs that are either too ambitious or too superficial, and is therefore another point in the planning process where formative work can be informative. For instance, our consideration to enable physical therapists to implement their guidelines within an acceptable time investment made us decide to use self-regulation. Another difficulty is that applying Intervention Mapping in program development is an iterative process so that program developers must have a certain degree of flexibility to learn from formative data, listen to the advisory committee, and backtrack as necessary.

Finally, the foregoing issues raised, indicate that thoroughly going through each step of Intervention Mapping may be a time consuming, and therefore costly process. It is indeed our opinion that familiarity and experience with the Intervention Mapping procedure may be a great advantage in this respect. The process may further benefit from sound planning based on anticipation on the subsequent steps. This can reduce the likelihood of unforeseen developments and too much backtracking, and consequently of the required time investment.

A positive, but challenging aspect of guidance provided by Intervention Mapping is that interventions be focused on various levels of the ecological model. In our case, this meant targeting both individual behaviour and practice level elements. We developed an intervention for guideline adherence of physical therapists, a factor that can be considered to be an environmental influence for patient behaviour and health outcomes. We also addressed quality managers, a group in the practice environment of the therapists. Our formative studies revealed several factors related to inadequate practice quality management that negatively influenced physical therapists’ guideline adherence. Although targeting these multilevel influences on low back pain care complicated our selection of behaviours, determinants, change methods and ultimately program components, our formative work provided us with a sound rationale for the development of our program.

## Conclusions

We conclude that, despite the difficulties we encountered, applying the framework of Intervention Mapping provided the required sound rationale for the development, implementation and evaluation of an intervention for the Dutch physical therapy CPG on low back pain based on multi method formative research. We expect that the stepwise approach of Intervention Mapping can be a valuable framework for future intervention development designed to improve guideline implementation. However, a decision aid to select determinants of guideline adherence identified in the formative research to analyse the problem may increase the efficiency of the application of the Intervention Mapping process.

### Ethical approval

The study was approved by the Committee on Medical Research Involving Human Subjects (CMO) Arnhem-Nijmegen (Filenr. CMO 2007/172).

## Abbreviations

GR: Geert M Rutten; JH: Janneke Harting; AS: Angelique Schlief; LKB: L Kay Bartholomew; RABO: Rob AB Oostendorp; NKV: Nanne K de Vries.

## Competing interests

The authors declare that they have no competing interests.

## Authors’ contributions

GR and JH conceived the study. Together with GR and JH, AS contributed to the formative research and the logistics of the study. LKB, RABO and NKV also contributed to the conceptual idea of the formative research and the intervention. All authors were involved in the development process of the intervention. GR drafted the manuscript, all other authors contributed to manuscript review and revision and read and approved the final manuscript.

## Supplementary Material

Additional file 1 Data sources and findings of the formative research. Click here for file
