# Supplementary material for: Development of a theory- and evidence-based intervention to enhance implementation of physical therapy guidelines for the management of low back pain
Source: Arch Public Health. 2014 Jan 15;72(1):1. doi: 10.1186/2049-3258-72-1 (PMC3897896; doi:10.1186/2049-3258-72-1)
Supplement: Additional file 1 — Data sources and findings of the formative research. [file 2049-3258-72-1-S1.doc]

Additional file 1: data sources and findings of the formative research

| STUDY | METHODS | RESULTS |
| --- | --- | --- |
| literature study  (Rutten & Rutten, 2004, unpublished) | - Conservative literature review - Medline, Embase, Psychinfo, The ERIC database, Cochrane library, references of publications - Mesh terms: diffusion of innovations; (practice) guideline; evidence based practice; attitude; self efficacy - Further search terms: implementation; innovation; dissemination; adoption; cognitions; meta cognition; social norm/social influence - Publications from 1990-2003 | - Various individual cognitive factors that were expected to relate to adherence with clinical practice guidelines. - Individual factors were mainly related to knowledge; attitude; social influences; some organizational factors - Hardly any publications that organized factors into behavioral determinants or constructs - Hardly any publications in which behavioral theories were applied. - Information specifically on factors related to implementation of physical therapy guidelines was limited to attitudes and skills |
| Qualitative  (Harting et al, 2009) | - 3 Focus group interviews with peer consultation groups - Semi-structured interview route based on theoretical framework - N = 30; 30% female - Practice experience varying from < 5 to > 30 years - Theoretical saturation - Qualitative content analysis with direct approach using NVivo 2.0 | **Knowledge**   - Negative tendency towards the guidelines - Limited interest due to passive dissemination strategy, disagreement with used terminology and extensiveness of the guideline - Misunderstandings about obligatory character of the guidelines   **Persuasion**   - Ambiguous attitudes due to doubts about credibility, advantage, lack of compatibility with patient’s demands, threat to professional autonomy, complexity and limited flexibility of the guideline - Perceived threats due to financial and organizational consequences   **Decision**   - Limited information about this stage - A negative perceived social norm to the use of the guideline   **Implementation**   - Limited information about this stage - Very limited application of the guideline due to forgoing aspects, a perceived lack of skills and perceived barriers concerning availability of measurement scales   **Confirmation/Maintenance**   - Very little information about this stage, due to a low number of ‘users’ - A perceived lack of facilitation by the professional organization |
| Quantitative  (Rutten et al, 2009) | - Cross sectional survey - Determinants and perceived adherence (questionnaires) and actual adherence (clinical vignettes judged at 6 recommendations) - N = 472; 49% female - Practice experience varying from < 5 to > 30 years - Analyses: descriptives for determinants and adherence; cross tabs for awareness; stepwise multiple regression analyses for relations between adherence and determinants; logistic regression for the relations between awareness and determinants | **Adherence**   - Mean percentage adherence = 50 %   **Awareness**   - 3 Awareness groups: realistic estimators (38.5%); over estimators (25.2%); under estimators (36.4%) - Awareness of personal performance interferes in the relation between determinants and adherence   **Relation between adherence and determinants**   - Adherence was related to the perceived relative advantage of the guideline, the attention paid to the guideline and the perceived absence of supportive facilitators   **Relation between determinants and misperceptions of personal adherence**   - Overestimation of personal adherence was mainly related to the perceived social norm and to perceived potential losses - Underestimation was related to the perceived social norm; the attention paid to the guideline and the perceived compatibility with current practice |
| GIPhT-study (2007/2010) | | |
| Literature study | - Conservative literature review - Focus on emotional and organizational factors of guideline adherence and factors for implementation and maintenance stages - Medline, Embase, Psychinfo, Cochrane library, references of publications - Mesh terms: diffusion of innovations; (practice) guideline; evidence based practice; attitude; self efficacy; emotions; organizational innovation; organizational culture - Further search terms: implementation; innovation; dissemination; adoption; cognitions; social norm/social influence; affective factors - Publications from 1990-2007 | - Hardly any publications found concerning emotional factors related to guideline implementation - Various emotional factors derived from behavioral theories, such as self respect/pride; confidence; shame/regret; guilt; annoyance/anger; discomfort - Various organizational factors related to innovation, such as role of manager; internal communication and structures; organizational size and change culture - Some individual level factors related to maintenance, such as positive attitude, high self efficacy, low perceived risks and high intentions. Most factors are on organizational/environmental level. - For physical therapy no publications found concerning emotional and/or organizational factors related to guideline implementation |
| Qualitative | - 4 Focus group interviews - 2 Groups with high adherence levels, 2 groups with mixed adherence levels - n = 29; 10% female - Practice experience varying from < 5 to > 30 years - Qualitative content analysis with direct approach using NVivo 2.0 | **Previous factors and practice**   - More information about previous practice; practice environment; innovativeness and felt needs and problems   **Knowledge**   - Ambiguous understandings about how to use the guideline and the possibility to diverge from their recommendations   **Persuasion**   - Findings of cognitive determinants comparable to interviews pilot study - Various emotional factors, such as fear, annoyance, satisfaction, confidence, pride, regret, guilt and discomfort   **Decision**   - Behavioral, social and tensional self efficacy, gathering information; trying out; trial by others   **Implementation**  *Individual factors*   - Negative perceived social norm and low perceived performance by others; high motivation to comply with patients’ preferences; insufficient competences and skills concerning yellow flags and outcome questionnaires; various barriers and forms of resistance   *Organizational factors: practice level*   - Practice structure: structural deliberation meetings; support management; creating opportunities; investing practice time; availability of materials - Practice culture: commitment towards guidelines; commitment towards practice achievements; respect; openness; innovativeness   *Organizational factors: professional organization level*   - Restructuring the practice field towards multidisciplinarity; performing implementation activities; supportive structuring of the professional group; culture change towards transparency; various forms of organizational resistances   **Confirmation/Maintenance**   - Still limited information about this stage, there appears to be an extensive overlap with factors for the implementation stage   *Individual factors:*   - Compatibility with other routines; feedback on adherence, outcomes and costs; various forms of reinforcement   *Organizational factors: practice level*   - Compatibility with practice aims and integration in practice routines; ongoing facilitation by practice management   *Organizational factors: professional organization level*   - Compatibility with professional organizations’ objectives and integration in professional organizations’ routines; ongoing facilitation by the professional organization |
| Quantitative | - Longitudinal survey: two measurement moments with 6 months time span - Determinants and perceived adherence (questionnaires) and actual adherence (clinical vignettes with 12 quality indicators) - N = 394; 44% female - Practice experience varying from < 5 to > 30 years - Analyses: descriptives for determinants and overall guideline adherence and individual quality indicator scores; paired samples T-test for differences at T0 and T1; cross tabs for awareness; bivariate correlations for relations between adherence and determinants; cross sectional and longitudinal multiple regression analyses for the complete group and for awareness subgroups; | **Adherence**   - Mean percentage adherence T0 = 49.2% and T1 = 49.9%, Differences of less that 2% between T0 and T1 mean scores on the individual quality indicators. Large differences of the various indicator scores, ranging from < 5% - > 95% - Lack of clinical reasoning skills; insufficient application of the ICF categories; lack of addressing of psychosocial factors; hardly any application of measurement instruments for health related patient outcomes   **Awareness**   - 3 Awareness groups over T0 and T1: realistic estimators (deliberative type; ±40%); over estimators (autonomous type; ±45%); under estimators (vigilant type; ±15%) - Over estimators perform better and score more favorable on various determinants than realists and under estimators - Awareness is mainly a moderating factor in the relation between determinants and guideline adherence   **Relation between determinants and adherence**  **Factors derived from cross sectional analyses**  *Individual factors:*   - Attention paid to the guideline; frequency of evaluation; flexibility; compatibility with current practice, potential losses of individual, motivation to comply social norm of colleagues and physicians, technical or cultural resistance; commitment, uncertainty about position; complexity - *Practice factors* - Structural deliberation, supportive practice culture; practice provides possibilities for retraining,   practice provides sufficient time for guideline adherent care  *Professional organization factors*   - Compatibility with professional organizations’ objectives ; supportive facilitators; restructuring practice field – providing means, integration in professional organizations’ routines   **Factors derived from longitudinal analyses**  *Individual factors:*   - Previous practice, relative advantage; visibility of results; feeling uncomfortable; tensional self efficacy; previous overestimation; flexibility and communicability of the guideline; uncertainty of therapist about position   *Practice factors*   - Supportive management in practice – structural deliberation; supportive practice culture   *Professional organization factors*   - Restructuring practice field - multidisciplinary arrangements; facilitative activities of the professional organization; market directed health care supports guideline adherence |
